# Acknowledgment to Reviewers of *Foods* in 2020

**DOI:** 10.3390/foods10020238

**Published:** 2021-01-25

**Authors:** Foods Editorial Office

**Affiliations:** MDPI AG, St. Alban-Anlage 66, 4052 Basel, Switzerland

Peer review is the driving force of journal development, and reviewers are gatekeepers who ensure that *Foods* maintains its standards for the high quality of its published papers. Thanks to the cooperation of our reviewers, in 2020, the median time to first decision was 14 days and the median time to publication was 33 days. The editors would like to express their sincere gratitude to the following reviewers for their precious time and dedication, regardless of whether the papers were finally published:
Aaslyng, Margit DallLiem, Djin GieAbate, GiuliaLila, Mary AnnAbbasi, Qammer H.Lim, Kar HoAbdollahi, MehdiLim, SangbinAbeele, Samuel VandenLin, Hai-ShuAbollino, OrnellaLin, Hong-Ting VictorAbugri, Daniel A.Lin, Hsin-TangAcedo, Jeella Z.Linares, Maria BelénAcevedo Fani, AlejandraLinares-Pastén, JavierAčkar, ĐurđicaLinderborg, KaisaAcquah, CalebLindholm Bøgh, KatrineAdamczak, LechLindsey, AlexAdamczyk, GretaLing, Ming-PeiAdedeji, AkinbodeLiou, Bo-KangAdhikari, AchyutLiou, Nai-ShangAdhikari, KoushikLipan, LeontinaAdiletta, GiuseppinaLiu, ChangqiAdjonu, RandyLiu, JianmingÅdnøy, TormodLiu, KeshunAdrover, AlessandraLiu, RongmingAfrin, SadiaLiu, YangAganovic, KemalLiu, YanhongAgerbirk, NielsLiu, YeyiAgòcs, AttilaLiu, ZhipengAgrimonti, CaterinaLizard, GérardAgriopoulou, SofiaLjubenkov, IvicaAgusa, TetsuroLo Scalzo, RobertoAgyei, DominicŁobacz, AdrianaAhn, SangdooLobo, GloriaAhn, Yeong HeeLockshin, LarryAhrens, Richard A.Łodyga-Chruścińska, ElżbietaAhrné, LiliaLogan, AmyAidi Wannes, WissemLogan, NatashaAiello, FrancescaLognay, Georges C.Aiello, GildaLohse, BarbaraAigotti, RiccardoLohumi, SantoshAires, AlfredoLoiseau, NicolasAit-Kaddour, AbderrahmaneLollier, VirginieAkıllıoğlu, GülLombardi, SilviaAlamillo, Josefa MuñozLombardo, MauroAlamprese, CristinaLomonaco, TommasoAlañon, ElenaLončarić, AnteAlashi, Adeola M.Longo, EdoardoAl-Asmar, AsmaaLopes, GracilianaAlba, VittorioLópez Nicolás, Jose ManuelAlba-Elías, FernandoLopez, ArnoldoAlbergamo, AmbroginaLopez, FrancescoAlberto Lopes, Joao FilipeLópez, MercedesAlbrecht, ElkeLópez, Miguel ÁngelAlcalde, Maria J.Lopez, RicardoAlegria, CarlaLopez, VictorAlencikiene, GitanaLópez-Galán, BelindaAlewijn, MartinLópez-García, GabrielAlexandri, MariaLópez-Gómez, José PabloAlexopoulos, AthanassiosLópez-Linares, Juan CarlosAl-Fatimi, MohamedLópez-López, AntonioAliakbarian, BaharLopitz Otsoa, FernandoAliaño González, María JoséŁopusiewicz, ŁukaszAlinovi, MarcelloLordan, RonanAljewicz, MarekLorenzo, Jose M.Alkasrawi, MalekLores, MartaAllaf, KarimLouarn, EssylltAllegra, AlessioLouppis, Artemis P.Allegret, Jean-PierreLoura, Luís M.S.Allegro, GianlucaLøvdal, TrondAlmajano, María PilarLoveridge, E. JoelAlmeida, João M.Low, JuliaAlmeida, Maria JuditeLozano-Ojalvo, DanielAlminger, MarieLu, ChunguiAlmli, Valerie LengardLu, ZhanhuiAloisi, IrisLubinska-Szczygeł, MartynaAltenbach, Susan B.Luca, Patrizia DeAltmann, Brianne A.Lucarini, MassimoAltosaar, IllimarLucci, PaoloAlvarenga, Nuno BartolomeuLucena, RafaelAlvarez, Juan B.Lücke, Friedrich-KarlÁlvarez-Mercado, Ana IsabelŁuczyńska, JoannaAlvarez-Suarez, JoseLudwiczak, AgnieszkaAlves, ElianaLudwig, DavidAmaro, Ana L.Lugo-Cervantes, Eugenia Del CarmenAmarowicz, RyszardLuís, ÂngeloAmato, MarioLukasiewicz, MarcinAmigo, Jose ManuelLukić, IgorAmigo, LourdesLukinac, JasminaAmmann, JeanineLuna-Vital, Diego A.Ammar Dilawar, MuhammadLung, Ming-YuAmores Arrocha, AntonioLunter, DominiqueAmoriello, TizianaLuo, XianAnastasaki, EiriniLupu, StelianAnastasio, AnielloLuridiana, SebastianoAndabaka, ŽeljkoLutfi, EsmailAndersen, Barbara VadLuti, SimoneAnderson, RobinŁysiak, GrzegorzAndo, MasashiM. Gonçalves, ElsaAndrade, IsabelM. Pedrosa, MercedesAndrade, María J.Macedo, AntóniaAndreescu, SilvanaMacia, AlbaAndreev, KonstantinMaciej, StrzemskiAndrić, FilipMaddock, RobertAnedda, RobertoMaehre, HanneAngelino, DonatoMaestri, ElenaAngeloni, GiuliaMafra, IsabelAngeloni, SimoneMaggi, FilippoAngkawijaya, Artik ElisaMagiatis, ProkopiosAnibal, JaimeMagni, ChiaraAnjos, RosárioMagri, GiuliaAnna, GrummonMagriplis, EmmanuelaAnnor, GeorgeMah, Jae-HyungAnsari, RaisMahato, NeelimaAnselma, LucaMahler, VeraAntczak-Chrobot, AnetaMaicas, SergiAntolak, HubertMaidannyk, ValentynAntonelli, AlbertoMaiorano, GiuseppeAntonelli, AndreaMaisch, TimAntonides, GerritMajchrzak, DorotaAntoniewska, AgataMajchrzak, TomaszAntonini, GiovanniMajeed, MohammedAntonissen, GuntherMajewska, Małgorzata P.Antonopoulou, IoMajolino, DomenicoAntunes, DulceMajumder, KaustavAntunovic, ZvonkoMäkinen, SariApea-Bah, Franklin BrianMakino, YoshioApetrei, ConstantinMaldonado-Valderrama, JuliaApolinar-Valiente, RafaelMaleknia, Simin D.Appell, MichaelMalet-Martino, MyriamAppenroth, Klaus JuergenMalfeito-Ferreira, ManuelAprea, EugenioMałgorzata, KowalskaAquilani, ChiaraMałgorzata, ZiarnoAquilanti, LuciaMalissiova, EleniAranda, AgustínMallikarjunan, KumarAraóz, RómuloMallor, CristinaArapitsas, PanagiotisMalmberg, PerAraujo, PedroMalvano, FrancescaArce, LourdesMamone, GianfrancoArczewska, MartaMan, SimonaAreal Borrego, FranciscoManary, Mark J.Arena, ElenaMancini, SimoneArena, KatiaMancuso, MoniqueAresta, AntonellaMandalari, GiuseppinaArgiriou, AnagnostisMandolesi, SerenaArioli, FrancescoManes, JordiAristizábal, Luis F.Mangalathu, SujithArosio, PaoloMangia, Nicoletta PasqualinaArrebola, Francisco JavierMangiagalli, MarcoArrowood, Michael J.Maniglia, BiancaArroyo-López, Francisco NoéMannina, LuisaArsiccio, AndreaMannino, GiuseppeArtieda, Diana AnsorenaMannu, AlbertoArunkumar, AbhiramMano, JunichiAsaro, FiorettaManousi, NataliaAscrizzi, RobertaMansour, RamziAshrafi, AmirmansoorManthey, Frank A.Aslam, MohamadMantzourani, IoannaAspée, Felipe JimenezMantzouridou, FaniAstruc, ThierryManuela, SannaAtanassova, MariaMao, YiminAthanasiadis, VassilisMaragou, Niki C.Atteritano, MarcoMaranghi, FrancescaAtuonwu, James C.MARANGON, MatteoAubourg, SantiagoMarangoni, AlejandroAugustyniak, AdrianMarchev, AndreyAussenac, ThierryMarchewka, JoannaAustin, ChrisMarciniak-Łukasiak, KatarzynaAvallone, SylvieMarconi, OmbrettaAvellone, GiuseppeMarek Sarić, MarijanaAverbeck, DietrichMarek, KieliszekAvi, ShpigelmanMargalef, MariaAvilés Ramírez, CarmenMargherita, AlessandroAvramidou, EvangeliaMargheritis, EleonoraAvula, BharathiMarhuenda, JavierAxiotis, EvangelosMaría José, JordánAykas, DidemMaria Teresa, FrangipaneAyora-Cañada, Maria JoseMariani, AngelaBa, YongMarina, CretenetBabini, ElenaMarina, KrvavicaBach, BenoitMarinangeli, Christopher P.F.Bach, KatrinMarini, FedericoBąchor, RemigiuszMariniello, LoredanaBączek, KatarzynaMarín-Iniesta, FulgencioBadanjak Sabolović, MarijaMarino, RosariaBadeka, AnastasiaMarino, TizianaBadiani, AnnaMarizzi, ChristineBaeza, ElisabethMarkiewicz-Keszycka, MariaBagchi, DebasisMarklinder, IngelaBagur-González, Maria GraciaMarković, KsenijaBahelka, IvanMarković, ZvjezdanaBai, JinheMarques, José C.Baiano, AntoniettaMarranzano, MarinaBaima, SimonaMarrone, GiuliaBaker-Austin, CraigMarrone, RafaelleBalan, PrabhuMars, Ruben A.Balasubramanian, BalamuralikrishnanMarsol, AlexisBalawejder, MaciejMartegani, EnzoBalayssac, StéphaneMarti, AlessandraBalcerek, MariaMartín García, DianaBaldisserotto, AnnaMartin, JenniferBall, ChristopherMartín, RafaelBall, PeterMartin, RoseBallester, JordiMartín-Cabrejas, Maria AngelesBalusamy, Sri RenukadeviMartindale, WayneBalzan, StefaniaMartin-Diana, Ana B.Balzaretti, Claudia MariaMartinez, Mario MartinezBamba, Bio Sigui BrunoMartínez, TomasBancalari, ElenaMartinez, VicenteBandari, SureshMartínez-Álvarez, ÓscarBandarra, Narcisa M.Martínez-Cañamero, MagdalenaBandyopadhyay, SmarakMartinez-Martos, Jose ManuelBanovic, MarijaMartínez-Monteagudo, Sergio I.Baranowska, HannaMartinez-Rios, VeronicaBarba, CarmenMartínez-Sanz, José MiguelBarbaric, MonikaMartínez-Villaluenga, CristinaBarbieri, SaraMartini, DanielaBarbosa, Joana Inês BastosMartino, CamilloBarbosa-Pereira, LetriciaMartino, GiuseppeBargmann, BasMartinotti, SimonaBarisic, LidijaMartins, Artur J.Baroň, MojmírMartins, CatiaBarreca, DavideMartín-Vertedor, DanielBarreca, FrancescoMärtlbauer, ErwinBarreca, SalvatoreMartuscelli, MariaBarreiro, Sonia LosadaMarzec, AgataBarrenetxea, Kizkitza InsaustiMasci, MaurizioBarresi, AntonelloMasci, StefaniaBarrientos Velazquez, Ana LuisaMashima, RyuichiBarroca, Maria JoãoMasino, FrancescaBarros, AnaMasotti, FabioBarros, AntonioMassad-Ivanir, NaamaBartolomeo, GiovanniMassaglia, StefanoBarton, LudekMassouras, TheofylaktosBarukcic, IrenaMastanjevic, KresimirBaruzzi, FedericoMastronardi, LuigiBasalekou, MarianthiMasucci, FeliciaBasiak, EwelinaMasullo, MariorosarioBasile, BorisMata, PaulinaBasile, TeodoraMatarneh, SulaimanBass, PhilMatei, FlorentinaBassani, AndreaMatencio, AdriánBastarrachea, Luis J.Mateo, JavierBasu, MeheliMateo, Jose J.Batista, PatríciaMaterska, MałgorzataBatkowska, JustynaMáthé, ÁkosBattistelli, NoemiMatiadis, DimitriosBavaro, Simona LuciaMatias, CatarinaBayer, KarlMatkowski, AdamBayliss, KirstyMatsakidou, AnthiaBazylko, AgnieszkaMatsuoka, HirokiBchir, BrahimMatt, MonikaBeauchamp, JonathanMatthew, BiwerBec, Krzysztof B.Matthews, Karl R.Beccaria, MarcoMattison, Christopher P.Bechoff, AurélieMatzzotti, FabioBecker, PierreMau, GunnarBednářová, MartinaMaugeri, Andrea GiuseppeBejaei, MasoumehMaurer, PatricBekhit, Alaa El-Din A.Mauro, VasconiBekiaris, GeorgiosMauromicale, GiovanniBelguesmia, YanathMavrommatis, AlexandrosBelka, MariuszMayumi, KoichiBellido-Milla, DoloresMazidi, MohsenBellissimo, NickMazurek, SylwesterBeltrán Sanahuja, AnaMazzeo, Maria FiorellaBeltran, GemmaMazzoni, ValerioBeltrán, José AntonioMazzucco, EleonoraBenalia, SourayaMcAnena, LiadhanBencardino, DanielaMcAuley, Catherine M.Bendini, AlessandraMcCarthy, HelenBenedé, SaraMcCarthy, Noel A.Benedek, CsillaMcgrath, SaraBenedetti, SimonaMcKay, FionaBengoechea, CarlosMcMillin, Kenneth W.Béni, SzabolcsMcSweeney, Matthew B.Benić, MiroslavMcVey, ClaireBenítez-Cabello, AntonioMeda, Naamwin-So-Bâwfu RomaricBenito, María J.Medana, ClaudioBenito-Román, ÓscarMedić, HelgaBenković, MajaMedina, SoniaBennett, BenMehariya, SanjeetBenohoud, MeryemMehli, LisbethBerenjian, AydinMejuto, JuanBerger, Ralf G.Melendez-Martinez, AntonioBerget, IngunnMelgar Castañeda, BrunoBergholz, TeresaMelilli, Maria GraziaBergman, ChristineMelini, FrancescaBerhow, Mark A.Melini, ValentinaBeriáin Apesteguía, María JoséMelis, RiccardoBerman, PaulaMenanteau-Ledouble, SimonBernacki, BruceMenčík, JaroslavBernaerts, TomMendes-Ferreira, AnaBernhard, ThuyMendonca, AubreyBerrada, HoudaMendum, RuthBerrue, FabriceMeneguzzo, FrancescoBertelli, DavideMenga, ValeriaBerthold-Pluta, AnnaMennella, IlarioBertoldo, MonicaMensuali-Sodi, AnnaBertram, HanneMenzel, CarolinBesada, CristinaMerah, OthmaneBesnard, GuillaumeMergenthaler, MarcusBetoret, NoeliaMerlino, Valentina MariaBetts, Natalie S.Mešić, ArminBeumer, RijkeltMesić, ZeljkaBever, CandaceMessia, Maria CristinaBevilacqua, AntonioMeyners, MichaelBeyrer, MichaelMezzasalma, ValerioBezirtzoglou, EugeniaMezzetti, BrunoBhandari, Manohar PrasadMhatre, EishaBhandari, Shiva RamMichal, KumštaBharath, Leena P.Michalak, IzabelaBhardwaj, HarbansMichalska, AnnaBhatta, SagarMichel, PiotrBher, AnibalMichel, VisalliBian, ZhonghuaMiddendorf, DanaBianchi, BiagioMidmore, DavidBianchi, GiuliaMiele, Nicoletta AntonellaBiancolillo, AlessandraMierzejewska, JolantaBiango-Daniels, MeganMierzwa, DominikBiasato, IlariaMigliore, GiuseppinaBibra, MohitMiguel, EugenioBierzuńska, PaulinaMikac, Lara M.Biesaga, MagdalegaMikkonen, KirsiBiesek, JakubMiklavcic, JohnBillaud, MarieMikołajczak, BeataBilska-Wilkosz, AnnaMikulski, DamianBimbo, FrancescoMikulski, DawidBinello, AriannaMilán-García, JuanBintsis, ThomasMilham, PaulBirgit, DekkersMillar, LynneBisko, Nina A.Miller, RhondaBittante, GiovanniMiller, VandanaBittsanszky, AndrasMillot, MarionBiziuk, Marek K.Milosavljević, IvanBlackmore, MarieMincione, AntonioBlackwell, BarbaraMinnelli, CristinaBlades, MabelMiraglia, DinoBlagojevic Zagorac, GordanaMiranda, JonatanBlaiotta, GiuseppeMiret Pastor, LluísBlanco, MireiaMironeasa, SilviaBlanco, PilarMirosa, MirandaBlanco-Salas, JoséMirzoian, ArmenBlandino, AnaMis Solval, Kevin E.Blando, FedericaMis, AntoniBlank, RalfMishima, TakashiBlasi, FrancescaMishra, AbhinavBłaszczak-Świątkiewicz, KatarzynaMishra, Amit KumarBlazevic, IvicaMishra, Kumud BandhuBleyaert, PeterMishyna, MaryiaBlissett, JacquelineMitakou, SofiaBlock, HushtonMitliagka, ParaskeviBlottière, HervéMitrus, MarcinBoatto, VascoMiyawaki, OsatoBobak, ŁukaszMiyazato, HironariBobbo, TaniaMladineo, IvonaBobowski, NualaMnif, WissemBoccia, Antonella CaterinaMoczkowska, MałgorzataBocianowski, JanMohamed, Azza H.Bogdanović, TanjaMohan, AnandBoggia, RaffaellaMohnen, DebraBogoni, PaoloMoldes, AnaBohrer, Benjamin M.Molin, MarianneBojić, MirzaMonaci, LindaBoles, JamesMonago-Maraña, OlgaBolhuis, DieuwerkeMoniruzzaman, MohammedBolumar, TomasMonks, JeniferBonaccorsi, IvanaMontaña, MaríaBonadonna, AlessandroMontanari, ChiaraBondoc, IonelMonteils, ValérieBondonno, Nicola P.Monteiro, FilipaBonos, EleftheriosMonteiro, Maria João PereiraBorchgrevink, CarlMontesano, CamillaBorczak, BarbaraMontesano, DomenicoBordiga, MatteoMontesinos-Pereira, DavidBornes, StéphanieMontevecchi, GiuseppeBoros, DanutaMontone, Carmela MariaBorowiec, Magdalena SłowikMontowska, MagdalenaBorra, DanielleMonzó, J. MartínezBos-Brouwers, HilkeMonzó-Cabrera, JuanBosch, ChristineMoon, BoKyungBoselli, EmanueleMoon, Kyoung-SikBosi, SaraMoore, Carla J.Bosilevac, JosephMoore, KarenBoskou, GeorgeMora, DiegoBošković, PericaMora, LeticiaBotelho, GoretiMoraga, JavierBotinestean, CristinaMorales, DiegoBottari, BenedettaMorales, Jose ManuelBotticella, ErmelindaMorante-Zarcero, SoniaBou, RicardMordenti, AttilioBoukid, FatmaMoreira, RamónBou-Maroun, EliasMorello, LauraBound, Sally A.Moreno, Antonio D.Bourlieu, ClaireMoreno, Francisco JavierBowker, Brian C.Moreno-Fernandez, JorgeBoy, ErickMoreno-García, JaimeBraca, AlessandraMoreno-Rojas, José ManuelBramanti, EmiliaMorfin, NuriaBranca, FerdinandoMorgan, SydneyBranciari, RaffaellaMorita, KyojiBrandt, KirstenMorkunas, IwonaBrasiello, AntonioMorozova, KseniaBrassard, JulieMorris, Craig F.Braud, AdelineMorris, GordonBravi, LauraMort, AndrewBravo, Francisca IsabelMorton, James D.Bravo-Diaz, CarlosMorzel, MartineBravo-Núñez, ÁngelaMoschopoulou, EkateriniBray, JeffMoscicki, LeszekBreidbach, AndreasMotoki, KosukeBrejnrod, AskerMourad, MarieBremer, PhilMourato, Miguel P.Brennan, CharlesMouritsen, OleBrereton, Paul A.Mourtzinos, IoannisBrezoiu, Ana-MariaMoyano, LourdesBrighenti, VirginiaMozzon, MassimoBrinson, RobertMu, RuipuBrkić Bubola, KarolinaMucalo, AnaBrnčić, Suzana RimacMuccilli, VeraBroadhurst, C. LeighMudura, ElenaBrodziak, AnetaMueller, GeoffreyBronkowska, MonikaMujaffar, SaheedaBrown, Paul B.Müller, CarstenBrown, Ronald B.Müller, KajetanBrugiapaglia, AlbertoMüller, Maren LilianBrühl, LudgerMulligan, ChristopherBruni, RenatoMultari, SalvatoreBryant, ChristopherMunafo, JohnBryła, MarcinMunekata, Paulo E.S.Bryla, PawelMuniz, JuanBrys, JoannaMuñoz-González, CarolinaBryszewska, Malgorzata AnitaMünstedt, KarstenBubola, MarijanMuramoto, KojiBuchanan, RobertMurmura, FedericaBuchtová, HanaMuroya, SusumuBuchweitz, MariaMusatti, AlidaBucić-Kojić, AnaMusioł, MartaBuckland, Nicola J.Mustafa, Ahmed M.Budroni, MarilenaMustafa, ArwaBudryn, GrażynaMusto, MauroBueno, MónicaMwihia, EvalynBuhr, Richard JeffMylona, PhotiniBuksa, KrzysztofNa, Chae-InBukvicki, DankaNabhan, GaryBurak, M. FurkanNadai, ChiaraBurdaspal, PedroNaderi, NassimBurke, AnthonyNagano, TakaoBursac Kovacevic, DanijelaNagappan, ArulkumarBursová, ŠárkaNaiker, ManiBusch, FlorianNair, DivekBustamante, María ÁngelesNajda, AgnieszkaButler, FrancisNakagawa, PabloButler, GillianNakakita, Shin-ichiByrd, James AllenNakamoto, HiroyukiCaballero, DanielNakamura, SoichiroCabrita, Maria JoaoNakano, KoheiCacak-Pietrzak, GrażynaNakashima, SouichiCacopardo, LudovicaNambidiyattil Govindan, ByjuCadavez, VascoNanno, MasanobuCahaner, AvigdorNaoki, FukumaCairrão, ElisaNapoli, Edoardo MarcoCais-Sokolińska, DorotaNarayan, RogerCalabrò, ViolaNarciso, Joan OñateCalado, RicardoNardi, TizianaCalanche, Juan B.Nardin, TizianaCalder, PhilipNarduzzi, LucaCalderon-Monge, EstherNarsimhan, GanesanCaldo, Kristian MarkNasheri, NedaCalín-Sánchez, ÁngelNatella, FaustaCallanan, Michael J.Nath, ArijitCalo-Mata, PilarNathanailides, CosmasCalvano, Cosima D.Natile, GiovanniCalvo Lerma, JoaquimNavarrete-Munoz, Eva MariaCamara, Jose SousaNavarro, CasildaCameron, SimonNavarro, Juan CarlosCamilli, EmanuelaNavarro, VirginiaCamillo, Angelo A.Navarro-Alarcon, MiguelCaminero, AlbertoNavarro-Hoyos, MirthaCamire, Mary EllenNavascués, EvaCammà, CesareNaviglio, DanieleCampa, ClaudineNawaz, Adil MalikCampbell, LydiaNawrocka, AgnieszkaCampisi, BarbaraNaznin, Most TaheraCampos, Camila D. MadeiraNazzaro, FilomenaCampos, DeboraNęcka, KrzysztofCampos, FranciscoNeffe-Skocińska, KatarzynaCampos, MariaNegro, CarmineCanon, FrancisNeilson, AndrewCantin, Celia M.Nenadis, NiknenCantini, ClaudioNepal, Mahesh RajCaparros Megido, RudyNetzel, MichaelCapasso, RaffaeleNeunert, GrazynaCaponio, FrancescoNewson, WilliamCaporaso, NicolaNexø, EbbaCaporizzi, RossellaNguyen, Khanh-HoangCapozzi, VittorioNguyen, Quang D.Cappa, CarolaNhung, Nguyen T.Cappelli, AlessioNiccolai, AlbertoCappelli, LucioNicolai, TacoCaputo, LeonardoNieddu, MariaCarballo, JavierNiedzielski, PrzemysławCarbone, KatyaNielsen, DaviaCarcea, MarinaNielsen, Søren Drud-HeydaryCardazzo, BarbaraNiero, GiovanniCardinali, AngelaNieva-Echevarría, BárbaraCardoso, SusanaNiimi, JunCardullo, NunzioNikbakht Nasrabadi, MaryamCaridi, AndreaNikkhah, AminCarini, EleonoraNikolaivits, EfstratiosCarins, JuliaNilsson, R. HenrikCarpenter, ElizabethNinčević Grassino, AntonelaCarpentieri, AndreaNinou, ElissavetCarradori, SimoneNioi, ClaudiaCarragher, JohnNiro, SerenaCarrascosa, ConradoNishi, Stephanie K.Carrera Fernández, CeferinoNishiyama, YusukeCarrera, MónicaNissen, LorenzoCarresi, CristinaNitride, ChiaraCarrillo, CeliaNobre, ClarisseCarroll, Kathryn A.Nochera, Carmen L.Carrubba, AlessandraNogales-Bueno, JulioCarter, KatharineNoguera Artiaga, LuisCarughi, AriannaNoordergraaf, Inge-WillemCaruso, GianlucaNorambuena, FernandoCarvalho, Ana Maria PintoNosch, Daniela SonjaCarvalho, DanielNosworthy, MatthewCasado, NataliaNovak Jovanovic, IvanaCasal, SusanaNovoselec, JosipCasanova, FedericoNovotni, DubravkaCasas Ferreira, Ana MaríaNowacka, MalgorzataCasassa, Luis FedericoNowak, AdrianaCasiello, GraziaNowak, AgnieszkaCastada, HardyNowak, DorotaCastanheira, IsabelNtaikou, IoannaCastellari, MassimoNtzimani, AthinaCastiglioni, BiancaNudda, AnnaCastillo, Manuel Z.Nunes, CeciliaCastrica, MartaNunes, CristianaCastro, Hélder F.Nunes, FernandoCastro, HelenaNuñez, AlbertoCastro-Muñoz, RobertoNunez, OscarCatalán, ÚrsulaNunziata, AngelinaCataldi., Tommaso R.I.Nwaiwu, OgueriCaterino, MariannaNychas, George-John E.Cattaneo, CamillaO’Callaghan, TomCautela, DomenicoO’Hair, JoshuaCavallo, CarlaO’Mahony, SeamusCavella, SilvanaO’Sullivan, MauriceCavoski, IvanaO’Sullivan, OrlaCayuela-Sánchez, José AntonioObeid, OmarCecchi, LorenzoObenland, DavidCeci, Luigi R.Ochmian, IreneuszCedeño, David L.Oehlke, KathleenCegielska-Radziejewska, RenataOfoegbu, Stanley UdochukwuCejudo Bastante, CristinaOgorek, RafalCelano, RitaOhm, Jae-BomCellini, AntonioOhnishi, KohtaCenci Goga, BeniaminoOhtsuka, AkiraCenteno, Juan A.Okińczyc, PiotrCerdá, BegoñaOkunade, Adewole L.Cerdà-Cuéllar, MartaOlafsdottir, GudrunCerqueira, LauraOlarte-Mantilla, SandraCerrato, AndreaOlejar, Kenneth J.Ceruso, MarinaOlejnik, AnnaCervantes, EmilioOlesen, Ann SofieCervelló, Gemma GutiérrezOliva, JoséCesa, StefaniaOliveira, AndreiaCevoli, ChiaraOliveira, HélderChacin-Suarez, AudryOliveira, JoanaChakrabarti, SubhadeepOliveira, José M.Chakraborty, RajaOliveira, SaraChalier, PascaleOliviero, TeresaChambel, LéliaOlszańska, Anna MariaChamberland, JulienOmar, Ahmad A.Chambers IV, EdgarOmar, Syed HarisChamlagain, BhawaniOmidian, KosarChan, Der-ShengOñate Narciso, JoanChan, Yu-YiOnchoke, KefaChandra-Hioe, Maria VeronicaOniszczuk, AnnaChang, Geng-RueiOniszczuk, TomaszChang, Te-ShengOnuh, John O.Chang, Tzu-ChengOnyeaka, HelenChang, YoonjeeOrabona, CirianaCharlebois, SylvainOracz, JoannaCharon, JustineOrdoudi, StellaChartier, AgnesOreopoulou, VasorChatzopoulou, PaschalinaOrkoula, MalvinaChaudhry, Muhammad Mudassir ArifOrkusz, AgnieszkaChaves-Lopez, ClemenciaOrlando, GiustinoCheel, JoséOrsburn, BenjaminChemat, FaridOrsini, MassimilianoChen, BernardOrtiz, CoralChen, GuibingOrtiz, DarwinChen, GuoxunOrtolá, María DoloresChen, HaotongOrtuño Casanova, JordiChen, Hsin-ChunOsawa, RoChen, Hung-ShingOscar, ThomasChen, Jen-TsungOsimani, AndreaChen, JiajiaOsivand, AsmaChen, LiliOstacolo, CarmineChen, Miaw-LingÖstbring, KarolinaaChen, Ming-HsuanÖstlund, SörenChen, XigaoOstos, Francisco JoséChen, Xuqi (Ricky)Ostrowska-Ligęza, EwaChen, YiliangOterhals, ÅgeChen, ZhaoOtlewska, AnnaCheng, Juei-TangOubiña, JavierCheng, Jya-WeiOun, AhmedCheng, Vincent Y-HOustric, PaulineCheng, Yuan-BinOvando-Martínez, MaribelChenoll, EmparOvissipour, MahmoudrezaChi, TingOwczarek, AleksandraChiabrando, ValentinaOwczarz, PiotrChiang, Hsin-IOzbay, GulnihalChiciudean, GabrielaOzeki, YasuhiroChiesa, Luca M.Pablos, FernandoChikayama, EisukePacetti, DeborahChilo, JoséPaciulli, MariaChinnici, FabioPadilla-Zakour, OlgaChiou, Tai-YingPagonopoulou, OlgaChira, KleopatraPaik, Hyun-DongChitarrini, GiuliaPaixao Coelho, Jose AugustoChitchumroonchokchai, ChureepornPająk, PaulinaChiva-Blanch, GemmaPajari, Anne-MariaChmiel, MartaPalanisamy, Uma DeviCho, Dong-WooPalermo, CarmenCho, SungeunPallottini, ValentinaCho, Young-EunPalma, Marco A.Chodak, IvanPalma, PierangelaChoi, In HagPalmeri, RosaChoi, Jong-ilPalmieri, GiannaChoi, Sung EunPalmieri, NadiaChoi, Young HaePampana, SilviaChoi, Young MinPanagou, Efstathios Z.Choi, Yun SangPanderi, IreneChow, SeinenPandino, GaetanoChristaki, EfterpiPanea, BegoñaChristofer Lendel, ChristoferPang, ShintaroChristoforides, EliasPanicz, RemigiuszChu, ChenggenPannella, GianfrancoChu, Tin-ChunPantano, PaulChul-sung, HuhPanzella, LuciaChung, Ching-HuPaolocci, FrancescoChung, Hyun-JungPaolucci, MarinaChung, Myong-SooPapadakis, GeorgeChung, SeojinPapadimitriou, KonstantinosChung, Yong SukPapadimitriou, VassilikiChung-Davidson, Yu-WenPapadopoulos, GeorgiosChuyen, Hoang V.Papadopoulou, OlgaCiaburro, GiuseppePapageorgiou, MariaCiardiello, Maria AntoniettaPapaioannou, EmmanouilCicatiello, ClaraPapale, MariaCiccoritti, RobertoPapamitsou, TheodoraCicero, ArrigoPaparella, AntonelloCicero, NicolaPapasotiropoulos, VasileiosCichelli, AngeloPapetti, AdeleCichowska, JoannaPapoti, Vassiliki T.Cidade, Maria TeresaPapoutsis, KonstantinosCieśla, JolantaPappalardo, GioacchinoCieślińska, AnnaPappas, ChristosCilla, AntonioPappas, Maria L.Cimini, AlessioParadiso, Vito MicheleCincotta, FabrizioParafati, LuciaCinelli, GiuseppeParamythiotis, SpirosCinq-Mars, DanyParaskeuas, Vasileios V.Cipak Gasparovic, AnaParaskevopoulou, AdamantiniCipolat, ClaudioPardo-Vicente, Miguel-ÁngelCirkovic-Velickovic, TanjaParente, EugenioCirrincione, SimonaParenti, OttaviaCiulu, MarcoParga-Dans, EvaCiurzyńska, AgnieszkaPark, ChulhwanCladis, DennisPark, Eun-YoungClanchy, FelixPark, Hyun JinClark, BethPark, Il-KwonClark, Natalie M.Park, Jonghun (Jay)Clarke, NeilPark, Ki HwanClaudia, Salanță LianaParlapani, Foteini F.Claus, HaraldParpinello, GiuseppinaClemente, AlfonsoParsons, BarryClementi, CatiaPaschino, PietroClericuzio, MarcoPaseiro-Cerrato, RafaelCliff, Margaret A.Pasias, Ioannis N.Clode, PetaPasinszki, TiborCocovi Solberg, David JaimePasławska, MartaCodacci-Pisanelli, GiovanniPasqualone, AntonellaCodina, GeorgianaPassos, Cláudia P.Coelho, AlfredoPastore, GianniCoelho, ChristianPatange, ApurvaCoker, Christine E. HarrisPatarata, LuísColantuono, AntonioPateiro, MirianColas, CyrilPatel, AshokColavita, GiampaoloPatenge, NadjaColle, MichaelPaterson, TonyColombo, Fabio MariaPathania, ShivaniColombo, Stefanie M.Patil, AnujaColonna, Maria AntoniettaPatz, Claus-DieterColoretti, FabioPau, Louis-FrancoisColquhoun, Thomas A.Paul, AmanCombes, Marie-christinePaul, Jean-YvesComino, PennyPaul, UttamCompagnone, DarioPaula, CorreiaConcas, Maria PinaPaulo Mirasol, EstherConcetta Valeria Lucia, GiosafattoPaulsen, PeterCondelli, NicolaPavli, FoteiniConesa, María R.Pavlovic, RadmilaConlon, MichaelPawelzik, ElkeConner, DavidPawlak, AleksandraConnors, NealPawlak-Lemańska, KatarzynaConte, AmaliaPawłat, JoannaConte, AngelaPazourek, JiříCook, Douglas R.Pecka-Kielb, EwaCooksey, KayPedan, VasilisaCordero-Bueso, GustavoPedonese, FrancescaCorella, DoloresPeira, GiovanniCornea-Cipcigan, MihaielaPeiretti, Pier GiorgioCorona, OnofrioPejcz, EwaCorrales-García, JoelPelin, MarcoCorreia, Ana CristinaPellegrino, LuisaCorreia, Carlos M.Pennanen, KyöstiCorreia, ManuelaPennisi, LucaCorsaro, CarmeloPennone, VincenzoCoscueta, EzequielPenumarti, AnushaCosentino, CarloPepe, TizianaCosme, FernandaPercival, Benita C.Cossignani, LinaPereira, AnaCosta, AntonellaPereira, CarlaCosta, Emilia MartinsPereira, Carlos DiasCosta, JoanaPereira, Cidália D.Costa, Maria Do CéuPereira, ElianaCosta, Nuno RicardoPereira, JorgeCostantini, AntonellaPereira, Jorge A.Costantini, LaraPereira, Laura M.Coulibaly, Wahauwouele HermannPereira, Ricardo N.Courtin, Christophe M.Pereira, VandaCozzolino, DanielPereira-Caro, GemaCrane, NathanPereira-Wilson, CristinaCrapo, CharlesPerera, ConradCravero, Maria CarlaPerestrelo, RosaCravotto, GiancarloPerestrelo, Rosa Maria De SáCremades Ugarte, JavierPérez Fernández, RaúlCrespi, StefanoPérez Navarro, JoséCreton, StuartPérez Palacios, TrinidadCristiana, PeanoPérez Puyana, Víctor ManuelCristina, RattiPerez, FelipeCrofton, EmilyPérez, María Del CarmenCropotova, JannaPerez, RaphaelCrowe, JessicaPérez-Álvarez, Jose AngelCrowe, WilliamPerez-Cano, FranciscoCruz, Rui M.S.Pérez-Correa, José RicardoCruz-Chamorro, IvanPerez-Cueto, ArmandoCuadrado, CarmenPérez-Gregorio, Maria RosaCudennec, BenoitPérez-Jiménez, JaraČukelj Mustač, NikolinaPérez-Lamela, ConcepciónCullere, MarcoPérez-Moral, NataliaCumbo, CosimoPeriago, Paula M.Cummins, AdrianPerino, SandrineCunha, Luís MiguelPerito, Maria AngelaCunha, SaraPerkins-Veazie, PenelopeCunningham, JudyPerpetuini, GiorgiaCuomo, FrancescaPerretti, GiuseppeĆurko, NatkaPerry, JenniferCvrtila, ZeljkaPerry, Michael J.Cwynar, PrzemyslawPersaud, KrishnaCybulska, JustynaPersky, SusanCzaplicki, SylwesterPersuric, ZeljkaCzarniecka-Skubina, EwaPerucka, IrenaCzech, HendrykPerujo, NuriaCzubaszek, AnnaPeschel, Anne O.Czyżowska, AgataPeters, John C.D’Ambrosio, MichelePetersen, Iben LykkeD’Amico, MarioPeterson, JuliaD’Amico, StefanoPetoumenou, DespoinaD’Antuono, IsabellaPetracci, MassimilianoD’Archivio, MassimoPetrakis, EleftheriosD’Ascenzi, CarloPetretto, GiacomoD’Errico, GerardinoPetronilho, SílviaD’Incecco, PaoloPetropoulos, SpyridonDa Silva Dias, Igor AlexandrePetrotos, KonstantinosDa Silva, A. MoreiraPetrozziello, MaurizioDa Silva, Anabela BernardesPetruzzi, LeonardoDa Silva, EricPeveri, ValentinaDadakova, EvaPexara, AndreanaDaems, DevinPezzolato, MarziaDaglia, MariaPfister, BarbaraDai, YuminPhan, Chin-SoonDalgaard, PawPhan, Minh Anh ThuDall’Acqua, StefanoPiacentini, EmmaDalle Zotte, AntonellaPiano, MartinaDalsgaard, Trine KastrupPiasecka-Kwiatkowska, DorotaDalzini, ElenaPiątkowska, EwaDambrosio, AngelaPicariello, GianlucaDanao, Mary-Grace C.Picchi, ValentinaDandekar, AdityaPicone, DeliaDandlen, Susana AnahiPicone, GianfrancoDando, RobinPiechowiak, TomaszDaniels, Mark J.Piecyk, MałgorzataDanielson, NeilPieczywek, Piotr M.Danikiewicz, WitoldPiekoszewski, WojciechDannenberger, DirkPielech-Przybylska, KatarzynaDapkevicius, MariaPiepiórka-Stepuk, JoannaDarewicz, MałgorzataPierce, Alex M.Darie, Costel C.Piergiovanni, Angela R.Daszkiewicz, TomaszPieroni, AndreaDávalos Herrera, AlbertoPieszka, MarekDaveran-Mingot, Marie-LinePignitter, MarcDavidovich-Pinhas, MayaPilolli, RosaDawidowicz, AndrzejPiluzza, GiannellaDawood, Mahmoud A.O.Piña Capó, Mª NievesDawson, PaulPina, MariaDay, Martin PeterPina-Perez, Maria ConsueloDe Angelis, ElisabettaPinedo-Rivilla, CristinaDe Boni, AnnalisaPinela, JoséDe Bruno, AlessandraPinent Armengol, MontserratDe Caro, PascalePinheiro, JoaquinaDe Clerck, CarolinePinheiro, LídiaDe Devitiis, BiagiaPinho, EvaDe Domenico, StefaniaPino, AlessandraDe Falco, BrunaPintado, TatianaDe Francesco, GiovanniPinto, LorisDe Kimpe, NorbertPinto, TeresaDe La Fuente Blanco, AranchaPiochi, MariaDe La Luz Cádiz-Gurrea, MaríaPiosik, JacekDe La Rosa, RaulPiotrowski, PaulinaDe La Torre, FernandoPires, EuclidesDe La Torre, XavierPirgozliev, VasilDe Leo, FedericaPirhonen, JuhaniDe Leo, VincenzoPisciotta, AntoninoDe Leonardis, AntonellaPiscopo, AmaliaDe Luca, GiuseppinaPistelli, LauraDe Lurdes Dapkevicius, MariaPittia, PaolaDe Masi, LuigiPizent, AlicaDe Melo, Marcelo M.R.Pizzolongo, FabianaDe Mendonça, Dina I.M.D.Planeta, DiegoDe Mieri, MariaPlaza, Encarna GomezDe Oliveira, Ivo VazPleadin, JelkaDe Rouck, GertPlessas, StavrosDe Santis, DianaPłotka-Wasylka, JustynaDe Sousa, Isabel Maria NunesPlows, JasmineDe Vargas Sansalvador, Isabel M. PerezPobiega, KatarzynaDe Wijk, Rene A.Poças, FátimaDean, Lisa OehrlPoczta-Wajda, AgnieszkaDebaste, FrédéricPodder, RajibDebonne, ElsPogorzelska-Nowicka, EwelinaDecker, Eric A.Poiana, MarcoDeeth, HiltonPolák, BeataDegani, GadPolak-Berecka, MagdalenaDegraeve, PascalPolari, JuanDe-Juan Vigaray, Maria DoloresPolenzani, BiancaDekker, MatthijsPolewski, KrzysztofDel Álamo Sanza, MariaPolidori, PaoloDel Bubba, MassimoPolišenská, IvanaDel Caro, AlessandraPolkinghorne, Benjamin G.Del Mar Campo, MaríaPołtowicz, KatarzynaDel Mar Romero, MaríaPomilio, FrancescoDel Nobile, Matteo AlessandroPongrac, PaulaDel Pozo Bayón, Maria A.Pons, AlexandreDel Río Celestino, MercedesPoór, MiklósDelattre, CédricPoortinga, Albert T.Delcenserie, VéroniquePop, Oana LeliaDeleu, LommePopa-Wagner, AurelDelfini, MaurizioPopovich, DeidreDelgado-Andrade, CristinaPoreda, AleksanderDelgado-Pando, GonzaloPorral, Cristina CalvoDell’Agli, MarioPorretta, SebastianoDella Malva, AntonellaPorrovecchio, AlessandroDellafiora, LucaPortanguen, StéphaneDeMarais, AlycePorto-Fett, Anna C.S.Deme, PragneyaPospiech, MatejDenes, Thomas G.Posri, WilatsanaDent, MajaPotaczek, Daniel P.Derewiaka, DorotaPotaniec, BartłomiejDermesonlouoglou, Efimia K.Potes, Maria EduardaDesdouits, MarionPotoroko, IrinaDesobry-Banon, SylviePouliot, YvesDesogus, FrancescoPower, AoifeDettlaff, KatarzynaPozo Sánchez, SantiagoDettori, Maria LuisaPozo, Elena PeñasDevasurendra, AmilaPrades, AlexiaDevesa, VicentaPradhan, AbaniDhungana, Sanjeev KumarPradhan, PrajalDi Bella, GiuseppaPrado-Cabrero, AlfonsoDi Cerbo, AlessandroPrandi, BarbaraDi Gioia, Maria LuisaPrasad, N. RrajendraDi Girolamo, RoccoPratap Singh, AnubhavDi Grigoli, AntoninoPrats, María SoledadDi Martino, PieraPravst, IgorDi Nunzio, MattiaPrazeres, AnaDi Pierro, ProsperoPreiner, DarkoDi Pinto, AngelaPrenzler, PaulDi Pizio, AntonellaPrester, LjerkaDi Rosa, Ambra R.Preston, TomDi Salle, AnnaPreti, RaffaellaDi Salvo, Martino LuigiPretorius, SakkieDi Venere, DonatoPrieto, CristinaDi Vita, GiuseppePrieto, Miguel ÁngelDias, Fernanda F.G.Prieto-Garcia, JoséDias, GorettyPrivat-Maldonado, AngelaDias, Luis G.Priyadarshini, MedhaDiaz, Jose ManuelProkopov, TsvetkoDíaz, María TeresaProroga, Yolande T.R.Diaz-maroto Hidalgo, María ConsueloProserpio, CristinaDíaz-Morcillo, AlejandroProtonotariou, StylianiDickinson, Harley D.Protudjer, Jennifer L.P.Didier, HauchardPrpić, ZvonimirDien, BrucePrusa, Kenneth J.Dietrich, Andrea M.Przybył, Jarosław L.Diezma, BelenPrzybylska-Balcerek, AnnaDifonzo, GrazianaPrzybylski, WiesławDijkstra, Albert J.Ptaszek, AnnaDikiy, AlexPtaszek, PawełDillon, Marina P. ArrietaPtaszynska, AnetaDimarogona, MariaPuchalska, PatrycjaDimitrellou, DimitraPuigdollers, CristinaDina, RăzvanPulina, PietroDing, XiongPulkrabova, JanaDini, IrenePulpytel, JeromeDinis, Lia Tânia J.Pummer, ElenaDinis, MárioPuolanne, EeroDinnella, CaterinaPurhagen, Jeanette K.Distefano, GaetanoPyatkovskyy, TarasDiuzheva, AlinaPycia, KarolinaDivis, PavelPyka, AlinaDjedaïni-Pilard, FlorenceQannari, El MostafaDludla, Phiwayinkosi V.Qazi, SohailDoctor, NinadQuadroni, SilviaDodero, VeronicaQuaglio, DeborahDomian, EwaQuain, DavidDomijan, KatarinaQuaresma, MárioDomínguez Díaz, LauraQuiñones, BeatrizDominguez, RubenQuintieri, LauraDomínguez-Álvarez, EnriqueQuinto, Emiliano JoséDomizio, PaolaQuinto, MaurizioDonadu, MatthewR Jaeger, SaraDonati Zeppa, SabrinaR. El-Seedi, HeshamDonno, DarioRaak, NorbertDoorenweerd, CamielRabadán, AdriánDop, Marie ClaudeRabbani, GulamDordevic, DaniRaber, JacobDorn-In, SamartRadwan, MohamedDorta, EvaRafiee-Tari, NiloufarDosoky, NouraRafińska, KatarzynaDossena, ArnaldoRago, DanielaDostalek, PavelRagucci, SaraDou, QingpingRagusa, AndreaDourtoglou, VassilisRaheem, BamideleDowns, KevinRaheem, DeleDoyen, AlainRahmani, DjamelDrabińska, NataliaRai, DilipDriessen, BertRai, RewaDrosinos, EleutheriosRajan, RobinDrzymała-Czyż, SławomiraRajendiran, KarthikrajDu Plessis, Heinrich W.Raju, RiteshDuarte, AmilcarRakic, VesnaDuarte, Vinícius D.Rakicka-Pustulka, MagdalenaDuda-Chodak, AleksandraRamanathan, PalaniappanDufossé, LaurentRamasamy, MohankandhasamyDugar, RohitRamirez, RosarioDulf, FranciscRamiro-Cortijo, DavidDumpler, JosephRamos, Inês N.Dunn, BruceRamos, MarinaDupont, Christopher M.Ramos, Patricia A.B.Durán-Guerrero, EnriqueRamos-Diaz, Jose MartinDurante, MirianaRanadheera, Chaminda SenakaDurazzo, AlessandraRanadheera, SenakaDürrschmid, KlausRanieri, MariannaDutta, PranabanandaRannou, CecilDvořáček, VáclavRanucci, DavidDzidic, AlenRanzato, EliaDziedzic, BarbaraRao, Niny Z.Dziekońska-Kubczak, UrszulaRao, ShiwangniDziewit, LukaszRapa, MattiaDziki, DariuszRaposo, AntónioDziuba, SzymonRasmussen, Torben SølbeckDzunkova, MariaRastrelli, LucaEady, PaulRato, Ana ElisaEbert, Timothy ARatusz, KatarzynaEconomou, VangelisRavallec, RozennEdwards-Callaway, LilyRawson, AshishEeckhaut, VenessaRayamajhee, VeeshanEfthimiadou, AspasiaRaynes, Jared K.Egan, DavidRaynie, DouglasEhrmann, Matthias A.Reale, AnnaEiden, GregoryRebollo-Hernanz, MiguelEidenberger, ThomasRecio, IsidraEilers, Elisabeth JohannaRecio-Menéndez, ManuelEkielski, AdamRecsetar, Matthew S.El Mecherfi, Kamel-EddineReddy, Chagam KoteswaraElansary, Hosam O.Regalado-González, CarlosElfalleh, WalidReguła, JulitaEl-Guendouz, SoukaïnaReinoso-Carvalho, FelipeElia, AntonioReis, AnaElias, MiguelRelitti, NicolaElida Nora, FerriRemize, FabienneElisa, De MarchiRemm, LiinaElisabetta, BraviRen, Yuan (David)Elkins, KellyRenaud, JustinELLIES, Marie-PierreRendueles, ManuelElls, TimothyRenna, MassimilianoElmassry, Moamen M.Rescigno, AntonioElmore, StephenResconi, Virginia CeliaEl-Seedi, HeshamRestuccia, DonatellaEncina-Zelada, Christian R.Revelou, Panagiota-KyriakiEndrizzi, IsabellaRevilla, EugenioEngevik, MelindaRevilla, IsabelEnglezos, VasileiosRezania, ShahabaldinEnjapoori, Ashwantha KumarRibaudo, GiovanniEnrico, PeterlungerRibeiro, AntonioErasmus, Sara WilhelminaRibeiro, Artur Jorge Araújo MagalhãesErlanson-Albertsson, CharlotteRibeiro, IsabelErler, SilvioRibeiro, Luís FilipeErmolina, IrinaRibeiro, Maria H.L.Escott, CarlosRibeiro, MiguelEscribano, Alfredo J.Ribeiro, VâniaEscudero, OlgaRibić, RosanaEskin, MichaelRicchi, MatteoEspada-Bellido, EstrellaRicci, AntonellaEspínola, FranciscoRicci, AriannaEsposito, FrancescoRichardson, Annette C.Esposto, SoniaRichardson, IanEssick, Greg K.Riera, BernardEsteve Mas, Maria JoséRighetti, LauraEsteves, EduardoRimac-Brnčić, SuzanaEstévez, MarioRinaldi, MassimilianoEstevinho, Berta Maria Abreu NogueiroRincon, Angela M.Esti, MarcoRincón, EstherEugenio, ApreaRipoll, GuillermoEuston, StephenRipolles-Avila, CarolinaEves, AnitaRistic, RenataExarhopoulos, StylianosRivaroli, SergioFabio, ZucconRivas, AnaFabiszewska, AgataRivas, SandraFaccia, MicheleRivero-Pérez, M. DoloresFadda, AngelaRizzello, Carlo GiuseppeFadda, CostantinoRizzi, ChiaraFagerlund, AnnetteRizzi, CorradoFaia, Arlete MendesRizzo, MilenaFailla, SebastianaRizzo, PaolaFalcão, Soraia I.Rizzo, ValeriaFalcone, Pasquale MassimilianoRobert, PazFaleiro, Maria LeonorRoberta, NuvoloniFallik, ElazarRobotti, ElisaFalqué López, ElenaRocchetti, GabrieleFan, RongRocha, Luiene M.Fan, XuetongRodov, VictorFancello, FrancescoRodrigo, LuisFanelli, Rosa MariaRodrigues, Ana SofiaFanelli, ValentinaRodrigues, HeberFantke, PeterRodrigues, MárcioFarahnaky, AsgarRodrigues, NunoFaraone, ImmacolataRodrigues, RuiFares, ClaraRodrigues, Sandra Sofia QuinteiroFaria, MiguelRodríguez Arcos, María RocíoFarina, VittorioRodríguez Bernaldo De Quirós, AnaFarkas, ÁgnesRodriguez Jovita, MarFarzaneh, VahidRodriguez Lagunas, Maria JoséFasolato, LucaRodríguez Villanueva, JavierFathi Abdallah, MohamedRodríguez, Armando AlexeiFauconneau, BenoitRodriguez, FernandoFauconnier, Marie LaureRodríguez, GuillermoFavati, FabioRodríguez, IsaacFavier, LidiaRodríguez-Calleja, Jose MaríaFecka, IzabelaRodríguez-Delgado, Miguel ÁngelFederighi, MichelRodriguez-Herrera, AlfonsoFeeney, EmmaRodríguez-López, PedroFelder, Robin A.Rodriguez-Lorenzo, LauraFelker, Frederick C.Rodríguez-Morató, JoseFeng, ChaohuiRodríguez-Solana, RaquelFeng, Chao-HuiRog, JoannaFengwei, XieRoger Philip, AidooFerguson, LouiseRoghair, Robert D.Ferlemi, Anastasia-VarvaraRohn, SaschaFernandes Lemos Junior, Wilson JoséRoig-Navarro, Antoni FrancescFernandes, AnaRokaya, MaanFernandes, Pedro A.R.Roleda, MichaelFernandes, TitoRomero, AlejandroFernández Barbero, GerardoRomero, GemaFernández Ochoa, ÁlvaroRomero, IreneFernández, AntonioRomero-González, RobertoFernández, ManuelaRoncarati, AlessandraFernandez, MariaRonda, FelicidadFernández, MarioRongai, DomenicoFernández, PabloRoose, GudrunFernandez, XavierRoosterman, DirkFernández-López, JuanaRoot, MartinFernández-Rodríguez, JavierRos Berruezo, GasparFernández-Soto, PedroRosa, AntonellaFernández-Tomé, SamuelRosa, MarcoFerrandino, AlessandraRoseland, JanetFerranti, PasqualeRosenberg, MosheFerraris, CinziaRosencrantz, Ruben R.Ferreira, IsabelRosenthal, AndrewFerreira, Nuno M.F.Rosicka-Kaczmarek, JustynaFerreira, Sónia S.Ros-Lis, JoseFerreira, SusanaRoss, AndrewFerreiro, MartaRoss, Robert L.Ferreiro-Vera, CarlosRossi, FilippoFerrer, EmiliaRossi, FrancaFerreri, CarlaRoszko, MarekFerri, MaurizioRotariu, LucianFerro, YveliseRoth, BjørnFeteira-Santos, RodrigoRothwell, Joseph A.Feveile Young, JetteRotondo, ArchimedeFeyissa, Aberham HailuRoudaut, GaelleFia, GiovannaRousseaux, SandrineFideline, Tchuenbou-MagaiaRoussis, Ioannis G.Figiel, AdamRoussis, VassiliosFigueira, Maria EduardoRoussos, Peter A.Figueiredo, Ana Cristina S.Rouster, PaulFigueiredo, Fernanda OtiliaRoy, DenisFilannino, PasqualeRoy, SwarupFilho, Luiz CarlosRóżycki, MirosławFilipello, VirginiaRóżyło, RenataFilipič, BratkoRubino, Federico MariaFilteau, MarieRubio-Hernández, Francisco J.Finglas, PaulRudzinska, MagdalenaFinocchiaro, FrancaRugman-Jones, Paul F.Finotti, EnricoRuiz Mendez, MariaFišera, MiroslavRuiz, LorenaFiszman, SusanaRuiz-Medina, AntonioFitsiou, EleniRumbos, ChristosFlahaut, ChristopheRumpunen, KimmoFlanjak, IvanaRuser, AlexanderFlemming, Hans-CurtRusinek, RobertFlorek, MariuszRusso, DanielaFlores-Félix, J.D.Russo, MarinaFlórez-Fernández, NoeliaRusso, PasqualeFlorowska, AnnaRustad, TuridFlorowski, TomaszRutherfurd-Markwick, KayFoddai, SebastianoRutigliano, MariacinziaFogarasi, MelindaRutkowska, JaroslawaFonseca, SoniaRutkowska, MałgorzataFonseca, Susana CaldasRybak, KatarzynaFont, RafaelRybicka, IgaFontecha, JavierRytel, ElżbietaForbes-Hernandez, TamaraRytkönen, PaulinaForghani, FereidounRyu, BomiFornal, EmiliaSabbah, MohammedFörst, PetraSabia, EmilioForth, Jan HendrikSacchi, RaffaeleForti, LucaSadati, MonirosadatFotakis, CharalambosSadhasivam, SudharsanFouladkhah, AliyarSadowska, AnnaFourny, NatachaSáenz Navajas, María PilarFoutch, Brian K.Sagnelli, DomenicoFowler, Ashley L.Saguer, ElenaFowler, StephanieSaha, SanjoyFox, Glen PatrickSaha, Sushanta KumarFraberger, VeraSahar, AmnaFracassetti, DanielaSahu, Saura C.Fragasso, MariagiovannaSaiano, FilippoFranceschi, PieroSaini, Ramesh KumarFrancis, LeighSaint-Eve, AnneFranco Ruiz, DanielSaiti, AnnaFranco, RafaelSaito, AkikoFrank, DamianSajdakowska, MartaFranke, KnutSakač, NikolaFrański, RafałSakai, NobuyukiFraqueza, MaríaSakkas, LambrosFratantonio, DeborahSalafranca Lázaro, Francisco J.Fratianni, AlessandraSalaheen, SerajusFravalo, PhilippeSaláková, AlenaFreire, M. SoniaSalas, Joaquín J.Friso, DarioSalas, José BlancoFroldi, GuglielminaSalaspuro, MikkoFrolova, YuliyaSaldo, JordiFrost, ScottSalek, Richardos NikolaosFu, XinyuSaliba, AnthonyFuentes, Màrius V.Salim, Nora Salina MdFukuda, KenjiSalis, AmandaFulignati, SaraSalles, ChristianFuochi, VirginiaSalovaara, HannuFusco, VincenzinaSalud, SerranoFusté-Forné, FrancescSaluti, GiorgioFuu, SheuSalvo, AndreaGabr, MoustafaSamaniego-Sánchez, CristinaGabriele, MorenaSamková, EvaGabriella, PasiniSammartino, Maria PiaGabrielli, PaoloSams, AnetteGaëlle, Pantin-SohierSamsonowicz, MariolaGagaoua, MohammedSánchez Ballesta, María TeresaGaglio, RaimondoSanchez Silva, AnaGago, CustódiaSánchez, Jesus LozanoGai, FrancescoSánchez-Gómez, RosarioGajdosik, Martina SrajerSánchez-Mata, María De CortesGajewska, DanutaSánchez-Ortiz, AraceliGál, RobertSánchez-Rodríguez, María IsabelGalani, Joseph Hubert YamdeuSanchiz, ÁfricaGalanis, AlexisSandhu, AmandeepGalante, EvaSängerlaub, SvenGałęcki, RemigiuszSanjeet, MehariyaGalego, LudovinaSanjeewa, Kalu Kapuge AsankaGalic, AnteSanmartin, ChiaraGalic, KataSannino, AnnaGałkowska, DorotaŠánová, PetraGallego, MartaSantagata, GabriellaGallen, CélineSantamaria, PietroGalli, ViolaSantana, AdinaGallina-Toschi, TulliaSantaoja, MinnaGallo, MonicaSanthakumar, Abishek BommannanGalus, SabinaSantiago, MichaelGambacorta, GiuseppeSantini, AntonelloGambaryan, StepanSantona, MarioGanatsios, VassiliosSantonicola, SerenaGancarz, MarekSantos, CarlaGao, JieSantos, Virgínia Alice Cruz DosGarber, Eric A.E.Santovito, ElisaGarbowska, MonikaSaravana, Periaswamy SivagnanamGarcés, VíctorSaravanakumar, KandasamyGarcia Estévez, IgnacioSardari, Roya R.R.Garcia Ivars, JorgeSardaro, Maria Luisa SavoGarcia, Coralia V.Šárka, EvženGarcia, CyrielleŠarkanj, BojanGarcia, M.R. InfantesSarriés, María VictoriaGarcía, Marta MesíasSarrocco, SabrinaGarcia-Álvarez-Coque, José-MariaSarshar, MeysamGarcía-Díez, JuanSasaki, KeisukeGarcia-Garcia, GuillermoSastry, SudhirGarcía-García, PedroŠatalić, ZvonimirGarcia-Gimenez, Maria DoloresSatish, LakkakulaGarcia-Gimeno, Rosa MariaSato, KenjiGarcía-González, ÁngelaSaucier, CedricGarcia-Gonzalez, NataliaSaurav, KumarGarcía-Herrera, PatriciaSaurina, JavierGarcia-Jares, CarmenSavard, ClaudiaGarcía-Molina, María DoloresSavell, JeffGarcía-Moreno, M. ValmeSavelli, ElisabettaGarcia-Moruno, EmiliaSavino, FrancescoGarcia-Sanchez, AsuncionSavoie, Jean-MichelGarcía-Segovia, PurificaciónSawicka, BarbaraGarcia-Vaquero, MarcoSawicki, TomaszGardeli, ChrysavgiSaxami, GeorgiaGareb, BahezSberveglieri, VeronicaGarima, KulshreshthaScaglioni, SilviaGarmyn, AndreaScampicchio, MatteoGarofulić, Ivona ElezScannell, Amalia G.M.Garrido-Fernández, AntónioScarafoni, AlessioGarrido-Maestu, AlejandroScarpellini, EmidioGartaula, GhanendraŠčetar, MarioGąsecka, AleksandraSchäfer, HannoGašić, Uroš M.Schauss, AlexanderGasparre, NicolaSchendel, RachelGast, Richard K.Schiavone, AchilleGaudette, Nicole J.Schieber, AndreasGaudichon, Claire C.Schiefermeier-Mach, NataliaGauer, JuliaSchifferstein, HendrikGavara, RafaelSchjøll, AlexanderGawalek, JolantaSchmidt, HeinarGawlik-Dziki, UrszulaSchmidt, Walter F.Gawroński, JacekSchmitz, OliverGazza, LauraSchnabel, UtaGebicki, JacekSchneider, FelicitasGeladi, PaulSchottroff, FelixGélinas, PierreSchoustra, Sijmen EGenovese, ClaudiaSchouteten, JoachimGenovese, FrancescoSchulz, FlorianGenovese, GiuseppaSchunko, ChristophGentile, AlessandraSchwarz, MónicaGentile, LuigiŚcibisz, IwonaGeorgiev, VasilScott, FrostGeorgiou, ConstantinosScotti, NunziaGerardi, CarmelaScuderi, AlessandroGerasopoulos, DimitriosŠebela, MarekGerbi, VincenzoSebranek, Joseph G.Gere, AttilaSeca, Ana Maria Loureiro DaGerique, AndrésSedlaříková, JanaGescher, AndreasSegatto, MarcoGetachew, Adane TilahunSegura Plaza, José FranciscoGetty, KellySeijo, M. CarmenGhanotakis, Demetrios DemetriosSeiquer, IsabelGharehyakheh, AminSękara, AgnieszkaGhazanfar, ShahinaSekiyama, YasuyoGhidini, SergioSelby-Pham, SophieGhidossi, RemySeleiman, Mahmoud F.Ghijsen, PaulSembratowicz, IwonaGhimire, BalkrishnaSenadeera, WijithaGhirardello, DanielaSendra, EstherGhoddusi, Hamid B.Sentandreu, EnriqueGholamipour-Shirazi, AzarmidokhtSentandreu, MiguelGhosh, AbhijitSeo, Han-SeokGhosh, SampatSeo, KunhoGiaccone, ValerioSeo, Kwon-ilGiacometti, JasminkaSeo, Sang WooGiamarchi, PhilippeSeraphin, DenisGiannakourou, Maria C.Serio, AnnalisaGiarratana, FilippoSerrà, AlbertGiavasis, IoannisSerra, AndreaGieczewska, KatarzynaSerradilla, Manuel JoaquínGikas, EvagelosSerralheiro, Maria LuísaGil, Maria ManuelSerrano-Pertierra, EstherGilissen, Luud J.W.J.Serreli, GabrieleGill, ChrisServenti, LucaGiménez-Gómez, PabloSestili, FrancescoGiovinazzo, GiovannaSettanni, LucaGiráldez, Francisco JavierSetyaningsih, WidiastutiGirard, AudreySevilimedu Veeravalli, SathyanarayananGiri, SibSevilla-Morán, BeatrizGiribaldi, MarziaSforza, StefanoGiromini, CarlottaSgorbini, BarbaraGisbert, Javier P.Shafiee, MojtabaGiuberti, GianlucaShakeri, MajidGiuffrè, Angelo MariaShamshiri, Redmond R.Giuggioli, Nicole RobertaShang, NanGiulia, ConversaSharma, ChetanGiurg, MirosławSharma, KavitaGiussani, BarbaraShehzad, AamirGlabska, DominikaSheikh, MehboobGlibowski, PawełShelley, MackGniewosz, MałgorzataShelomi, MatanGo, Gwang-WoongShen, CangliangGodoy, Bibiana JuanShen, YangGoedegebure, Robert P.G.Sheng, RuilongGogo, Elisha OtienoShete, VarshaGoicoechea, EncarnaciónShimada, Ken-ichiroGołaś, IwonaShimizu, TaroGołębiowski, MarekShin, Jung-AhGoliomytis, MichaelShipley, PaulGomes, Ana T.P.C.Shoji, ToshihikoGomes, SóniaShpigelman, AviGomez Roldan, Maria VictoriaSiano, FrancescoGómez, InmaculadaŠic Žlabur, JanaGómez-Alonso, SergioSicuro, BenedettoGómez-Laguna, JaimeSidari, RossanaGómez-Plaza, EncarnaSiegel, AmandaGonçalves, Elsa MargaridaSienkiewicz, MonikaGonçalves, Fernando J.Sierra, IsabelGonçalves, TâniaSikes, Anita L.Gonda, SandorSikirzhytski, VitaliGondek, EwaSikora, ElżbietaGonkowski, SlawomirSikorska, EwaGonzales-Barron, UrsulaSikorsky, MarekGonzález Martín, María InmaculadaŠilha, DavidGonzalez Viejo, ClaudiaSilva, CarlaGonzalez, John MichaelSilva, Catarina L.Gonzalez-Barreiro, CarmenSilva, Célia C.G.Gonzalez-Casado, AntonioSilva, FilomenaGonzález-García, JorgeSilva, Juan L.González-Gil, Esther M.Silva, Marcelo SousaGonzález-Miret, M. LourdesSilva, MarisaGonzález-Palacios, SandraSilva, PaulaGonzález-Paramás, Ana M.Silva, PedroGonzález-Redondo, PedroSilva, Severiano R.Goodman, Richard E.Silvério, Sara C.Goolaup, SandhiyaSilvetti, TizianaGordillo Arrobas, BelénŠimat, VidaGori, AntonellaŠimek, PetrGorinstein, ShelaSimó-Alfonso, Ernesto FranciscóGorjanović, StanislavaSimón Magro, EdurneGornas, PawelSimonato, BarbaraGoroncy, AlexanderSimone, VincenziGórska, AgataSimones Grilo, FilipaGórska, SabinaSimonne, AmaratGórska-Warsewicz, HannaŞimşek, ŞenayGorynski, KrzysztofSinanoglou, VasiliaGoto, TatsuhikoSingh, DeoGoto-Inoue, NaokoSingh, JashbirGovaris, AlexanderSingh, RajbirGraber, Zachary T.Singh, ReemaGrabska, JustynaSingh, Rupesh KumarGraça, CarlaSiotto, MariacristinaGracia, AzucenaSipiczki, MatthiasGradziel, Thomas M.Sipos, LászlóGraikou, KonstantiaSipos, PéterGrajewski, JanSisto, FrancescaGramza-Michałowska, AnnaSitkowska, BeataGranato, DanielSiudem, PawełGranica, SebastianSivertsen, Hanne KristineGrassi, SilviaSkalicky, MilanGrasso, SimonaSkenderidis, ProdromosGrauwet, TaraSkendi, AdrianaGray, TimSkonberg, DeniseGreatrex, Ben W.Skowron, KrzysztofGreco, ClaudioSkruzny, MichalGrembecka, MałgorzataSkrypnik, Liubov N.Grieco, FrancescoSkuland, Silje ElisabethGrigorakis, KritonSlaghenaufi, DavideGrillo, GiorgioŚliżewska, KatarzynaGrob, KoniŚlusarczyk, SylwesterGrohmann, LutzSmeriglio, AntonellaGrönman, KaisaSmiljanić, KatarinaGrossi Bovi Karatay, GrazieleSmit, BartGrossmann, ManfredSmith, Mark A.Grosso, ClaraSmits, TimGrosso, GiuseppeSmolensky, DmitriyGrunert, Klaus G.Smoluk-Sikorska, JoannaGrunow, BiankaSmrke, SamoGruszczyńska, JoannaSmutný, TomášGrygier, AnnaSmykal, PetrGrynkiewicz, GrzegorzSnopek, LukasGryszkin, ArturSobeh, MansourGryta, MarekSobocińska, MagdalenaGrzyb, JoannaSobota, AldonaGuaita, MassimoSocas-Rodríguez, BárbaraGuamis, BuenaventuraSogari, GiovanniGuan, VivienneSokół-Łętowska, AnnaGuedes, Cristina MirandaSolah, Vicky A.Guedes-Alonso, RaycoSolanki, Manoj KumarGuerra, ManuelaSolano-Aguilar, Gloria IsabelGuerra, Nelson PérezSoliño, LucíaGuerreiro, AdrianaSolomakos, NikolaosGuerreiro, Catarina SousaSołowiej, BartoszGuerrieri, NicolettaSolval, Kevin MisGuerrini, LorenzoSomma, StefaniaGuers, John J.Son, AhjeongGugołek, AndrzejSon, Yang-JuGuichard, ElisabethSone, IzumiGuidetti, RiccardoSonowal, HimangshuGuidi, AlessandraSontag-Strohm, TuulaGuido, LuisSoRelle, Elliott DanielGuil-Guerrero, José LuisSoriano, José MiguelGuillamón, EvaSorrentino, AlidaGuillén, Maria DoloresSortino, GiuseppeGuillén-Bejarano, RafaelSosnowska, DorotaGuiné, RaquelSoto, ErnestoGuiso, MarcellaSõukand, RenataGumienna, MałgorzataSoundharrajan, IlavenilGunaje, JayaramaSovova, HelenaGunaratne, NadeeshaSpadaccini, RobertaGupta, CharuSpano, GiuseppeGuriec, NathalieSpeciale, AntonioGushina, IrinaSpence, CharlesGussoni, MaristellaSpence, MichelleGustaw, WaldemarSperlì, GiancarloGuthausen, GiselaSpiegler, VerenaGutierrez, SalvadorSpínola, VítorGutiérrez-Gamboa, GastónSplichal, IgorGutkowska, KrystynaSplichalova, AllaGuzek, DominikaSprague, MatthewGuzman, CarlosSqueo, GiacomoGuzmán, EduardoŚrednicka-Tober, DominikaGuzman, IvetteSroka, ZbigniewGuzmán, PaulaStadnik, JoannaGyémánt, GyöngyiStan, LauraGyörgy, BázárStaniek, HalinaHa, Jae-WonStanzer, DamirHa, MinhStarowicz, MałgorzataHaag, AndreasStarzak, KarolinaHaba, HamadaStasa, PavelHaber, Lynne T.Stasiak, Dariusz M.Hackl, ThomasStasiak-Różańska, LidiaHać-Szymańczuk, ElżbietaStasiewicz, Matthew J.Hadinezhad, MehriStavolone, LiviaHaenlein, George F.W.Stefanelli, PatriziaHaertlé, ThomasStefania, PucciarelliHaghiri, MortezaStefanucci, AzzurraHalagarda, MichałSteffen-Heins, AnjaHalford, Nigel G.Stein, Alexander J.Haliński, Łukasz P.Steinhoff, UlrichHallmann, EwelinaStella, SimoneHam, Jun-SangStender, Emil G. P.Hamidi-Asl, EzatStepanic, VisnjaHamlin, RobertStepien, AnnaHammer, KatherineStępniowska, AnnaHan, DongWoonSterczyńska, MonikaHan, Sung GuSteup, MartinHan, ZhenlinStiburkova, BlankaHanaka, AgnieszkaStirke, ArunasHanania, MichelStocco, GiorgiaHandorf, OliverStojakowska, AnnaHanga, Mariana Petronela (Petra)Stout, JeffreyHanning, IreneStowers, Kristen CookseyHannisdal, RitaStraatman, Anthony G.Hano, ChristopheStranieri, StefanellaHano, TakeshiStrati, Irini F.Hantusch, BrigitteStrauss, JohannesHanus-Fajerska, EwaStromberg, ZacharyHappel, AustinStrube, JochenHarasym, JoannaStrzemski, MaciejHarbertson, JamesStudzinska-Sroka, ElzbietaHardacre, AllanStuetz, WolfgangHarnedy, Pádraigín A.Stuper-Szablewska, KingaHaro, DiegoStushnoff, CecilHaro, IsabelSu, DanHarper, JamesSubaric, DragoHarrison, SabineSubedi, DineshHasegawa, MorifumiSubedi, KiranHasegawa, YukiSubramani Paranthaman, BalasubramaniHasoňová, LucieSubramanian, ParthasarathiHassan, Sherif T.S.Succi, MariantoniettaHatano, TsutomuSuchanek, Mateusz .Hatzakis, EmmanuelSugimoto, YukioHatzdimitriou, EffieSuh, Hyung JooHawkins, Leigh K.Suh, JoonhyukHe, FeiSujak, AgnieszkaHe, JieSujka, MonikaHeber, DavidSuleria, Hafiz Ansar RasulHebishy, EssamSułkowska-Ziaja, KatarzynaHeide, MortenSuman, MicheleHelena Siegel, MarciaSummer, AndreaHeleno, SandrinaSummo, CarmineHellwig, MichaelSun Hong, JungHempel, GünterSun, YvonneHenchion, MaeveSung, JeehyeHenen, MorkosSung, Wen-ChiehHenriques, Ana RitaSuomela, Jukka-PekkaHeredia-Guerrero, José AlejandroSuzzi, GiovannaHernández Pérez, Maria Del PilarSvec, IvanHernández, FranciscaŠvecová, BlankaHernández, María Del MarSvoronos, Paris D.N.Hernandez, Maria JesusSwaney-Stueve, MarianneHernández-Borges, JavierSweedler, JonathanHernández-Hernández, OswaldoSweeney, Mary RoseHernández-Ledesma, BlancaŚwiątkiewicz, MałgorzataHernández-Mesa, MaykelŚwiderski, FranciszekHernández-Sánchez, NataliaŚwieca, MichałHernando, IsabelŚwiergosz, TomaszHerppich, WernerSych, JaniceHerraiz, TomásSýkora, JanHerrera-Herrera, AntonioSynytsya, AndriyHerrero, HernándezSzablewski, TomaszHerring, Andy D.Szalay, LaszloHertel, ChristianSzaleniec, MaciejHIjikata, AtsushiSzczepanek, JoannaHill, AnnieSzekalska, MartaHill, Arthur R.Szeleszczuk, ŁukaszHill, SandraSzendrő, KatalinHinrichs, JörgSzewczyk, KatarzynaHirotaka, KanedaSzpyrka, EwaHlisnikovský, LukášSzulc, KarolinaHo, KacieSzulc, PiotrHochegger, RupertSzultka-Młyńska, MałgorzataHocquette, Jean-FrançoisSzumny, AntoniHodges, TylerSzweda, PiotrHoffmann, Klaus H.Szwengiel, ArturHogan, SeanSzymandera-Buszka, KrystynaHogg, TimSzymanek, MariuszHohenstein, Jessica D.Szymanowska, UrszulaHolalu, Srinidhi V.Tabanelli, GiuliaHolman, BenjaminTabler, TomHong, Geun-PyoTaboada-Rodríguez, AmauryHong, Kwon-hoTabtabaei, SolmazHopfer, HeleneTaddei, FedericaHopp, ManuelTadesse, WuletawHoracek, MichaTagele, Setu BazieHorhat, Florin GeorgeTagliaferri, SaraHornedo-Ortega, RuthTagliazucchi, DavideHorvat, DanielaTaguchi, SeiichiHosomi, RyotaTahara, YusukeHosoyamada, MakotoTahergorabi, RezaHou, QinfuTaibi, AmelHoward, LukeTaiti, CosimoHoy, Mary KatherineTakahashi, KoretaroHrbek, VojtechTakayuki, TsukuiHrynko, IzabelaTakeda, FumiomiHsieh, Chang-WeiTakemura, KenshinHsieh, Lu-ShengTakenaka, ShinjiHsieh, Yun-Hwa PeggyTalavera, MartinHsieh, YvesTalens, ClaraHsu, Fong-FuTalon, RegineHu, BifengTamburino, RacheleHu, ChenlinTamsyn, StanboroughHu, YaxiTan, ChenHuang, ShuanglongTan, LiboHuang, Tung-ShiTan, SongwenHuang, XinTanabe, KatsuyukiHuang, Ya-lingTanaka, TakujiHuck, ChristianTaneyo Saa, Danielle LaureHuerta, NelsonTang Siah Ying, PatrickHuertas, Jesús R.Tang, JumingHugel, SylvainTangeland, TorvaldHulánková, RadkaTaniguchi, MoyuHume, Michael E.Tankiewicz, MaciejHung, Chin-ChuanTao, FeifeiHur, Sun JinTaoka, YousukeHurst, PhilTaoukis, PetrosHussain, HidayatTappi, SilviaHwang, Daniel Liang-DarTarabella, AngelaHwang, Kwon TackTarko, TomaszHyun, Sunghyup SeanTarola, Anna MariaIaccheri, EleonoraTarr, GarthIacopetta, DomenicoTarrago, LionelIacumin, LucillaTarragon Cros, ErnestoIametti, StefaniaTassoni, AnnalisaIammarino, MarcoTassou, ChrysoulaIanieri, AdrianaTate, RothwelleIannetti, LuigiTateo, FernandoIanni, FedericaTatjana, StevanovicIbáñez, ElenaTavaniello, SiriaIbáñez, Rodrigo A.Taviano, Maria FernandaIbrahim, SalamTawfike, AhmedIentile, RiccardoTaylor, MatthewIglesias-Carres, LisardTe Pas, MarinusIgne, BenoîtTedeschi, GabriellaIgual, MartaTedeschi, TulliaIliada, LappaTeichert, JoannaInam Afzal, MuhammadTeixeira, AlfredoInge, Van DammeTeixeira, NatérciaIngrassia, MarziaTellez-Lance, AngelaInguglia, Elena SofiaTello, JavierInomata, TakenoriTemerdashev, Zaual A..Inturri, RosannaTemesi, ÁgostonIoannis, K. KarabagiasTen Have, GabriellaIoannou, IrinaTena, NoeliaIorizzo, MassimoTenore, GianIqdiam, BasheerTerpou, AntoniaIrakli, MariaTersey, Sarah A.Iriondo, AmaiaTesson, VincentIrrera, NatashaTeter, AnneIsani, GloriaTheradiyil Sukumaran, AnurajIseppi, LucaThilakarathna, Surangi H.Iseppi, RamonaThomaidis, Nikolaos S.Islam, Mohammad ZahirulThomareis, ApostolosIslam, ShahidulThomas, RaymondIsobe, TomohikoThomas, WayneItabashi, YutakaThompson-Witrick, Katherine A.Ito, HideyukiThurner, ThomasIturimendi, NereaTian, BinIwaniak, AnnaTiano, LucaIwasaki, TomohitoTidona, FlavioJackowski, KarolTilley, MichaelJackson-Davis, ArmitraTintaru, AuraJacquier, Jean-ChristopheTinti, AnnaJahandideh, ForoughTirado, Diego F.Jahncke, MichaelTirelli, AntonioJain, AnshikaTirloni, EricaJaiswal, AmitTittarelli, FabriziaJakiela, SlawomirTIXIER, Anne-SylvieJakobek, LidijaTodd, Ewen C.D.Jakobusic Brala, CvijetaTodolí, José LuisJakubczyk, AnnaToepel, UlrikeJakubczyk, EwaTofalo, RosannaJakubczyk, KarolinaToldrá, FidelJakubowski, MarekTollner, Ernest W.Jalil, Abbe Maleyki MhdTolve, RobertaJanczarek, MonikaTomaniova, MonikaJanda, KatarzynaTomasello, BarbaraJang, Hae WonTomasevic, IgorJaniszewska-Turak, EmiliaTomasino, ElizabethJankowska, MilenaTomczyk, MichalJansen, WiebkeTomic, NikolaJanssens, KimTomkin, GeraldJara Palacios, María JoséTomonaga, ShozoJaradat, NidalTomoskozi, SandorJarończyk, MałgorzataTonolo, FedericaJaros, DorisToppe, JogeirJastrzębska, AnetaTor, MarcJatkowska, NataliaTornesello, Anna LuciaJaworska, DanutaTörök, ÁronJedziniak, PiotrTorres Perez, María DoloresJefferies, Laura K.Torres, IrinaJeleń, Henryk H.Torres, NazarethJemrić, TomislavTorri, LuisaJensen, Ida-JohanneTorrico, Damir D.Jensen, Jørgen DejgårdTorrieri, ElenaJeppesen, Per BendixTosi, PaolaJerkowić, IgorTouraki, MariaJeromel, AnaTouraud, DidierJeruszka-Bielak, MartaTovar, Clara Asuncion RodriguezJeżewska-Zychowicz, MarzenaTovar, JuscelinoJi, ShaofeiTraffano-Schiffo, María VictoriaJia, LiliTrafiałek, JoannaJiménez Cantizano, AnaTrani, AntonioJiménez Carvelo, Ana MaríaTremblay, FrancoisJiménez-Araujo, AnaTremlová, BohuslavaJimenez-Flores, RafaelTremonte, PatrizioJin, ZhaoTres, AlbaJo, CheorunTrevisani, MarcelloJoão Ramalhosa, MariaTrif, MonicaJohnson, ErikTrifol, JonJohnson, RonaldTrinh, Kieu The LoanJokić, StelaTripodi, GianlucaJonathan B., CooperTriviño-Tarradas, PaulaJones, J. AndrewTrombetta, MariaJones, Jessica L.Tropea Garzia, Giovanna Maria DanielaJones, Owen GriffithTrudo, SabrinaJones, StephenTrujillo, Antonio-JoséJones, VerityTrujillo-Cayado, LuisJoo, Seon-TeaTruzzi, CristinaJordão, António ManuelTrystram, GillesJosé Manuel, Perea MuñozTrzaskowska, MonikaJoseph, LucyTsafantakis, NikolaosJourdes, MichaelTsai, Po-JungJoy, MargalidaTsai, Shu-YaoJózsa, LászlóTsai, Yung-HsiangJóźwik, ArturTsimidou, MariaJuan García, CristinaTsironi, TheofaniaJuárez Varón, DavidTsochatzis, Emmanouil D.Jukić Špika, MajaTsopelas, FotisJukić, MarkoTsopmo, ApollinaireJun, Joon-YoungTsoupras, AlexandrosJung, SamooelTsutsumi, TomoakiJungnickel, ChristianTu, Hung-PinJuśkiewicz, JerzyTubaro, FrancoJuszczak, LesławTufarelli, VincenzoJuvik, John A.Tufariello, MariaJůzl, MiroslavTufariello, MiriamKabutey, AbrahamTůma, ZdeněkKachrimanidou, VasilikiTundis, RosaKafarski, PawełTunick, MichaelKagawa, NatsukoTurrini, AidaKageyama, HakutoTurrini, FedericaKahlon, Talwinder S.Tyl, Catrin E.Kalina, MichalTyler, RobertKalisz, AndrzejTylewicz, UrszulaKalisz, StanisławTyliszczak, BożenaKalit, Milna TudorTyuftin, Andrey A.Kallas, ZeinTzanavaras, Paraskevas D.Källback, PatrikTzanova, MilenaKalle, RaivoUbeda, CristinaKallen, VictorUchiyama, TaketoKallithraka, StamatinaUdenigwe, Chibuike C.Kalogianni, DespinaUdomkun, PatchimapornKalogiouri, NatasaUeda, HiroshiKam, RothmanUeno, ShigeakiKamenidou, IreneUgliano, MaurizioKamienik, JosefUhlen, Anne KjerstiKamiloglu, SenemUkic, SimeKamperman, TomUllah, IrfanKana, AntoninUlusoy, BeyzaKanauchi, MakotoUmerska, AnitaKanda, AtsushiUnderhill, Steven J.R.Kandylis, PanagiotisUniacke-Lowe, ThérèseKaneda, IsamuUnno, KeikoKaneko, GenUpadhyay, Rakesh K.Kang, Hui-SeungUrlich, SebastianKantono, KevinUsai, MariannaKapetanakou, AnastasiaUsuki, ToyonobuKappel, NoemiUtsuzawa, ShinKapusta, IreneuszValcarcel, JesusKarabagias, IoannisValdés García, ArantzazuKaragiannis, TomValdez, JoseKaramonova, LudmilaValenti, BernardoKarasawa, KenValentini, PaolaKaravoltsos, SotiriosValenzuela, Juan LuisKarcz, DariuszValero Diaz, AntonioKarim, AzharulValero, DanielKarlović, SvenValero, Javier SanzKarlsson, Anders H.Valero, ManuelKarnaouri, AnthiVallès, JoanKarpak, BirsenValli, EnricoKartheek, AnekellaValoppi, FabioKarwowska, MałgorzataVan De Walle, DavyKasapidou, EleniVan Den Heever, Johan P.Kassama, LaminVan Der Schaaf, UlrikeKatashima, TakuyaVan Eenennaam, Alison LKatayama, ShigeruVan Itterbeeck, JoostKate M., ThomasVan Poucke, ChristofKathleen, LacyVan Raaij, FredKatikou, PanagiotaVan Sanford, DavidKayano, Shin-ichiVan Vuuren, Hennie J.J.Kebede, BiniamVandeweyer, DriesKelly, PhilVapaatalo, HeikkiKemper, JoyaVarelas, VassileiosKendall, HelenVarga, LászlóKeppler, JuliaVargas-Murga, LilianaKershaw, JonathanVarzakas, TheodorosKęska, PaulinaVastola, AntonellaKetnawa, SunanthaVaz, Josiana A.Ketta, MohamedVazquez Espinosa, MercedesKevany, KathleenVázquez-Araújo, LauraKhachik, FredVega Gutierrez, SarathKhadka, ManojVega-Zamora, ManuelaKhan, AyubVeiga, MarianaKhan, NaimVelasco-Torrijos, TrinidadKharbach, MouradVelázquez Blázquez, José SebastiánKhattab, TawfikVelickovic, DusanKhmelinskii, IgorVella, Filomena MonicaKhurana, VarunVemula, HarikaKicel, AgnieszkaVemuri, RavichandraKiczorowska, BożenaVendrell, JosepKieliszek, MarekVenema, PaulKierończyk, BartoszVenter, CarinaKilcawley, KieranVenturi, FrancescaKilian, KrzysztofVenturi, ManuelKilonzo-Nthenge, AgnesVera Rodríguez, ManelKim, ChyerVera, PaulaKim, Gap-DonVerardo, VitoKim, Hae-YeongVergara, DanieleKim, Hyun-JinVerkempinck, SarahKim, In-JungVerlinden, BertKim, Jae KwangVerma, Anil K.Kim, Jang EokVermeir, IrisKim, Ji YeonVerneau, FabioKim, Jong-SangVerni, MichelaKim, JunheonVernoux, Jean-PaulKim, Kwang-OkVerrez-Bagnis, VéroniqueKim, LouVerruck, SilvaniKim, Mi-jeongVersari, AndreaKim, Nam HoonVetvicka, VaclavKim, Sang SookViapiana, AgnieszkaKim, Seung-HyunVibeke, OrlienKim, Soo RinVicente, LauraKim, Soo-KiVickery, KarenKim, WookiVidaček, SanjaKim, YeonsooVideau, PatrickKim, Young-JunVidic, JasminaKim, YoungmiVidrih, RajkoKim, Yun-baeViegas, OlgaKimbaris, AthanasiosVieira, Elsa F.Kinchla, AmandaVieira-Pinto, MadalenaKing, ChristianViejo, Claudia GonzalezKing, Joan M.Vierck, KellyKinobe, RobertViganò, RobertoKiokias, Sotirios N.Vignali, GiuseppeKiss, AttilaViktorova, JitkaKita, AgnieszkaVila, NataliaKitaoka, SatoshiVilarinho, FernandaKittler, SophieVilariño, NataliaKiyama, RyoitiVilela, AliceKlassen, RolandVilela, SofiaKleekayai, ThanyapornVilla, CaterinaKlewicka, ElzbietaVillalba, MayteKlir, ZeljkaVillalon, MarinaKljak, KristinaVillanueva, MarinaKljusurić, Jasenka GajdošVillar, Josep M.Kmiecik, DominikVillaverde, Juan JoséKnaapila, AnttiVillorbina, GemmaKnittelfelder, OskarVincent, AntonyKnudsen, Knud Erik BachVincenzo, De LeoKnupp, GerdVinci, GiulianaKo, Wen-ChingVinha, AnaKobayakawa, TatsuVirto, MailoKobayashi, MakotoViscecchia, RosariaKobus, ZbigniewVisioli, FrancescoKoch, Stefanie A.J.Vitale, LucaKoch, WojciechVitali Čepo, DubravkaKocherbitov, VitalyViuda-Martos, ManuelKocsubé, SándorVivar-Quintana, Ana M.Koehler, S. EleonoreVoća, NevenKoga, ShioriVoca, SandraKoh, EunmiVolpe, Maria GraziaKöhler, KarstenVolpe, Richard JamesKohyama, NorikoVon Wallbrunn, ChristianKoirala, RanjanVon Wright, AtteKókai, ZoltánVrchotová, NadêždaKokkinakis, Emmanouil N.Vriesekoop, FrankKokoska, LadislavVrzal, TomášKokoszyński, DariuszVukušić Pavičić, TomislavaKokotkiewicz, AdamVyskočil, VlastimilKoksel, FilizWackerlig, JudithKołodziejczak, MałgorzataWagle, Basanta RajKolodziejczyk, JoannaWagner, LianeKołożyn-Krajewska, DanutaWaite-Cusic, Joy G.Komprda, TomášWakayama, MamoruKondyli, EfthymiaWakayama, MasatakaKong, LingyanWałecka-Zacharska, EwaKong, XiangruiWales, AndrewKonno, NaotakeWall, MarisaKonopelski, PiotrWan, ZifanKonopka, IwonaWanasundara, JanithaKontogiannatos, DimitriosWang, Be-JenKontogiorgis, ChristosWang, BoKontominas, Michael G.Wang, Huei-JuKontoudakis, NikolaosWang, Jee-ChingKopeć, AnetaWang, JinKopel, PavelWang, JunqiKopjar, MirelaWang, LiaoKoppel, KadriWang, Myeong-HyeonKopsahelis, NikolaosWang, Pei-MingKordowska-Wiater, MonikaWang, PenghaoKorona-Glowniak, IzabelaWang, Qian JaniceKortesniemi, MaariaWang, QinKorzeniowska, MalgorzataWang, ShaohuaKos, BlaženkaWang, WeicangKos, IvicaWang, XinyuKoschella, AndreasWang, YanwenKosmala, MonikaWang, Yi-ChengKostadinovic Velickovska, SanjaWang, YifeiKostecka-Gugala, AnnaWang, ZhihanKostić, Aleksandar Ž.Wangchuk, PhurpaKot, AnnaWarriner, KeithKot, Anna MariaWaszkowiak, KatarzynaKotlowski, RomanWatanabe, MikiKotowicz, DawnWaterman, CarrieKotowicz, MarekWatkins, Peter J.Koubaa, MohamedWatrelot, Aude AnnieKoukounaras, AthanasiosWawrzyniak, AgataKoulman, AlbertWawrzyniak, JolantaKoumoto, KazuyaWazed, Md AbdulKouřimská, LenkaWeber, KonradinKourkoutas, IoannisWeckx, StefanKoutelidakis, Antonios E.Wee, JosephineKovačević Ganić, KarinWegren, Stephen K.Kovács, ZoltánWeidmann-Desroche, StéphanieKowalczewski, PrzemysławWeiner, MyraKowalczyk, DariuszWeisstein, Fei L.Kowalczyk, KrzysztofWendin, KarinKowalik, JaroslawWendy, WismerKowalska, HannaWessel, Dulcineia F.Kowalska, KatarzynaWesseler, JustusKowalski, RadosławWestad, FrankKozačinski, LidijaWhalen, Elizabeth A.Kozłowicz, KatarzynaWhent, MonicaKrafft, ChristophWianowska, DorotaKraska, ThorstenWicker, LouiseKraśniewska, KarolinaWicklund, TrudeKrause, TobiasWieczorek, PiotrKrause, TorstenWiege, BertholdKrauze-Baranowska, MiroslawaWiehle, MartinKrier, FrançoisWierzbicka, ElżbietaKrisch, JuditWiesman, ZeevKrischek, CarstenWietstock, PhilipKristo, AleksandraWiktor, ArturKroc, MagdalenaWilde, H. DaytonKról, JolantaWilde, Peter J.Krstanović, VinkoWilkins, Charles L.Krupa-Kozak, UrszulaWilkinson, KerryKruszewska, JoannaWillenberg, InaKtenioudaki, AnastasiaWilson, Jeffrey M.Ku, Kang-MoWilson, PeterKu, SeockmoWilson, ThomasKubiak, PiotrWiniarska-Mieczan, AnnaKubo, IzumiWinkler-Moser, Jill K.Kučera, LukášWińska, KatarzynaKuczyńska, BeataWinterfeld, AndreeaKueneman, EricWise, PaulKukula-Koch, WirginiaWismer, Wendy V.Kulawik, PiotrWitczak, MariuszKulczyński, BartoszWitrick, KatherineKulig, DominikaWitte, WolfgangKulig, RyszardWiwart, MarianKulis, MichaelWłodarczyk, MaciejKulma, MartinWłodarczyk-Stasiak, MarzenaKulozik, UlrichWłodarska, KatarzynaKumar, AnandWójciak, KarolinaKumar, JitendraWójciak, MagdalenaKumar, PankajWojciechowicz-Budzisz, AgataKumar, VikasWojdyło, AnetaKunicka-Styczyńska, AlinaWojnar, WeronikaKuo, Meng-I (Marie)Wojnowski, WojciechKuorwel, KuorwelWojtasik-Kalinowska, IwonaKupka, TeobaldWojtunik-Kulesza, Karolina A.Kurasiak-Popowska, DanutaWojtusik, MateuszKurdziel, MagdalenaWołejko, ElżbietaKurek, MiaWołosiak, RafałKuri, VictorWong, MauriceKurihara, HideyukiWoo, Shih-LungKurzawa, MarzannaWood, JeffKus, PiotrWood, KevinKusec, GoranWooding, StephenKushkevych, IvanWöstemeyer, JohannesKüster, InesWouters, ArnoKuzdraliński, AdamWoźniak, ŁukaszKuźma, ŁukaszWright, MalcolmKwak, Hyo SunWrona, MagdalenaKwasniewski, MishaWroniak, MałgorzataKwon, Jung-HwanWronkowska, MałgorzataL’Hocine, LamiaWu, JiandongLaaksonen, OskarWu, Jong-ShinnLabes, AntjeWu, RuhaiLabrosse, RoxaneWu, Sz-JieLabrou, NikolaosWunderlich, Shahla M.Lacarra, Teresa GarcíaWuyts, SanderLachenmeier, DirkXi, LinLachman, JaromirXiao, YongshengLachowicz, SabinaXiao, ZhenleiLaezza, AntonioXie, ZhuohongLaganà, Valentina RosaXiong, JiaLalas, StavrosXu, JianLaleg, KarimaXu, JieLamacchia, CarmelaXuan, Tran DangLambe, NicolaXynias, Ioannis N.Lammers, Volker R.G.Yamashita, MichiakiLampi, Anna-MaijaYan, WeikaiLamy, ElsaYáñez, RemediosLandi, MarcoYang, Angela Wei HongLange, EwaYang, BoLangeland, MarkusYang, CrystalLangiano, ElisaYang, GuangLangowski, Horst-ChristianYang, Kai MinLangridge, PeterYang, Ray-YuLangrish, TimYang, ShiLanotte, LucaYang, XianqinLanza, MassimilianoYang, YingLaPointe, GisèleYanni, Amalia E.Lappa, IliadaYano, JunyaLaranjo, MartaYasuda, ShinLaratta, BrunaYe, LiyunLarder, Christina E.Ye, ZhiweiLasik-Kurdyś, MałgorzataYedjou, Clement G.Latif, SajidYee, Callista StephanieLatino, Maria ElenaYeh, Ching-HuaLau, TiffanyYehualaeshet, TeshomeLaukkanen-Ninios, RiikkaYen, Feng-LinLaureys, DavidYeum, Kyung-JinLauro, Maria RosariaYeung, LeoLavelli, VeraYoo, Hyun JuLavilla, MariaYoo, Ji YounLawley, MeredithYoo, Michelle Ji YeonLe Bourvellec, CarineYoon, Ki-SunLe Dréan, GwenolaYoung, Howard A.Le Quéré, Jean-LucYu, Hsu-ShengLe Thanh-Blicharz, JoannaYuann, Jeu-Ming P.Le, Anh V.Yucel, UmutLê, SébastienZabetakis, IoannisLeao, JoséZachariadis, GeorgeLeber, BettinaZádor, FerencLebrón, José AntonioZadravec, ManuelaLech, KrzysztofZagklis, DimitrisLeclercq, LoïcZagrodzki, PawełLee, Chang JooZajac, MarzenaLee, Dong-UnZakłos-Szyda, MalgorzataLee, Hwa-JinZamaratskaia, GaliaLee, HyungjaeZambon, AlessandroLee, JeehyunZamostny, PetrLee, Jeong SangZanardi, EmanuelaLee, JihyunZand, NazaninLee, Pui YeeŽanetić, MirellaLee, Sang GilZanini, BarbaraLee, SanghyunZárate, RafaelLee, SangmiZarea, Richard N.Lee, Se HeeZarkada, AnnaLee, Sonny T.M.Zarrelli, ArmandoLegetic, BrankaZaukuu, John-Lewis ZiniaLegua, PilarZavrel, TomasLehmann, UndineZawada, KatarzynaLehtonen, Mari I.Zawadzki, PrzemysławLeisner, Jørgen J.Żbikowska, AnnaLeitner, ErichZdarta, AgataLeitner, GabrielZdolec, NevijoLeiva-Brondo, MiguelZdybel, EwaLemmens, ElienZeeshan, MuhammadLemming, Eva WarensjöZeppa, GiuseppeLeni, GiuliaZerva, AnastasiaLenoir, IreneZettel, ViktoriaLeon, LorenzoZhang, GuanshiLeong, Sze YingZhang, JunzengLeoni, ClaudiaZhang, KaiLeoni, ValerioZhang, XingminLeontopoulos, StefanosZhang, YiLeporini, MariarosariaZhang, ZhiyunLerma, Joaquim CalvoZhao, JingLerno, Larry A.Zhao, XihongLeroy, FredericZhao, YanLesmes, UriZheng, HaotianLesnik, JulieZhong, YongpingLestringant, PaulineZhu, ChangfuLetcher, ToddZiarno, MałgorzataLevaj, BrankaZielinska, DanutaLévesque, LucZielińska, DorotaLevrino, Gustavo A. MariaZielińska, EwelinaLewandowicz, GrazynaZieniuk, BartłomiejLewandowicz, JacekZimmermann, BorisLewis, MikeZimoch-Korzycka, AnnaLi, MengmengZinnai, AngelaLi, MiZiobro, RafałLi, Po-HsienZiogas, VasileiosLi, QiongyuZipori, IsaacLi, SijingZitz, UlrikeLi, WeiZiuzina, DanaLi, XuehuiZłotek, UrszulaLi, YanZnamirowska, AgataLi, YonghuiZoric, ZoranLianou, AlexandraZou, WeiLiao, Hung-JuZoumpopoulou, GeorgiaLiao, Yu-ChiehZoumpoulakis, PanagiotisLiaw, Chih-ChuangZovko Končić, MarijanaLibera, JustynaŽučko, JuricaLiburdi, KatiaZukiewicz-Sobczak, WiolettaLichtenberg-Kraag, BirgitŻulewska, JustynaLicon, CarmenZunin, Paola

